# Long-Term Clinical and Ecological Impact of an Antimicrobial Stewardship Program on the Incidence of Carbapenem-Resistant *Klebsiella pneumoniae* Infections in a High-Endemic Hospital

**DOI:** 10.3390/antibiotics13090792

**Published:** 2024-08-23

**Authors:** Teresa López-Viñau, Montserrat Muñoz-Rosa, Lidia Mª Ruiz-Lara, Lucrecia García-Martínez, Isabel Machuca, Irene Gracia-Ahufinger, Rafael Ruiz Montero, Juan José Castón, Ángela Cano, Elisa Ruiz-Arabi, José Ramón del Prado, Inmaculada Salcedo, Luis Martínez-Martínez, Julián Torre-Cisneros

**Affiliations:** 1 Pharmacy Unit, Reina Sofia University Hospital, 14004 Cordoba, Spain; 2 Infectious Diseases Unit, Reina Sofia University Hospital, Maimonides. Biomedical Research Institute of Cordoba (IMIBIC), University of Cordoba (UCO), 14004 Cordoba, Spain; 3 Centro de investigación Biomédica en Red de Enfermedades Infecciosas (CIBERINFEC), Instituto de Salud Carlos III, 28029 Madrid, Spain; 4 Microbiology Unit, Reina Sofia University Hospital, Maimonides. Biomedical Research Institute of Cordoba (IMIBIC), 14004 Cordoba, Spain; 5 Department of Agricultural Chemistry, Edafology and Microbiology, University of Cordoba (UCO), 14004 Cordoba, Spain; 6 Preventive Medicine and Public Health Unit, Reina Sofia University Hospital, Maimonides. Biomedical Research Institute of Cordoba (IMIBIC), University of Cordoba (UCO), 14004 Cordoba, Spain

**Keywords:** antimicrobial stewardship program, carbapenems, antimicrobial resistance, carbapenem-resistant *Klebsiella pneumoniae*, carbapenem-resistant infections, intervention

## Abstract

Carbapenem-resistant *Klebsiella pneumoniae* (CR-Kp) is currently a serious global concern. Antimicrobial stewardship programs (ASPs) are one of the key strategies to overcome this resistance. However, evidence about the long-term clinical and ecological impacts of ASPs is scarce. A multidisciplinary team conducted a multifaceted intervention in a CR-Kp endemic hospital over a 6-year period. We assessed the monthly long-term impacts of ASPs on carbapenem use, incidence density (ID), and crude death rates of hospital-acquired CR-Kp infections. Other variables potentially related to CR-Kp incidence and healthcare activity indicators were monitored. Carbapenem use showed a sustained reduction over the long term, with a difference of −66.19% (95% CI −87.03 to −45.34) between the expected pre-intervention trend consumption value and that obtained six years after starting the program. The ID of CR-Kp also decreased significantly and was maintained over the long term, with a relative reduction of −88.14% (95% CI; −100.4 to −75.85) at the end of the study period. The crude death rate of CR-Kp at 14 and 28 days decreased significantly after the intervention and remained steady after six years. Infection control indicator trends remained stable. This mixed ASP contributed to reducing the high incidence of infections and mortality rates of CR-Kp, achieving a sustained ecological and clinical effect.

## 1. Introduction

Carbapenem-resistant Gram-negative bacilli (CR-GNB) are currently a serious global concern [1]. In Spain, these microorganisms have increased considerably in recent years, especially carbapenem-resistant *Klebsiella pneumoniae* (CR-Kp), with an increasing trend in the number of isolates [2]. These strains can acquire different resistance mechanisms that prevent the use of not only carbapenems but also aminoglycosides, fluoroquinolones, colistin, and even more recently ceftazidime avibactam [3,4,5]. As a result, invasive infections are difficult to treat and are associated with high mortality [6]. Although there are several causes that promote antimicrobial resistance, the overuse of antimicrobials is decisive. This, coupled with the current scarcity of effective therapeutic options, has led the scientific community and public health agencies to urgently call for the rational use of available antibiotics [7,8]. Antimicrobial stewardship programs (ASPs) have proven to be a valuable tool to optimize antimicrobial use, and their implementation in healthcare facilities has increased exponentially in recent years. However, we still lack solid evidence to demonstrate which interventions can achieve clinical and ecological benefits, especially when focusing on specific multidrug-resistant phenotypes such as CR-Kp [9,10,11]. This is due, firstly, to the great heterogeneity in the methodological quality of most studies, and only a small number of these provide microbiological data, with great variability in the outcome endpoints [12,13,14,15,16]. Moreover, most studies have only demonstrated short-term effects [12,16,17] and were performed in settings without the endemicity of CR-Enterobacterales [18,19]; so, the impact in highly endemic areas is uncertain. 

Since 2014, our hospital has implemented an ongoing institutional Program for the Validation of Restricted-Use Antibiotics (PROVAUR) aimed at carbapenems in response to the high rate of *K. pneumoniae*-producing KPC-3 (KPC-Kp, a class A carbapenemase), that our center has suffered since an outbreak started in June 2012 [20]. These carbapenemases are vehiculized by plasmids that encode other resistance determinants, thus creating a profile of multidrug resistance with few therapeutic options.

PROVAUR showed a positive effect two years after its implementation in reducing carbapenem use and the incidence of nosocomial infections/colonizations caused by CR-GNB [21]. However, the effect of these programs tends to attenuate over time, especially those that incorporate a restrictive measure, as in our case. With this work, we aim to evaluate the long-term impact of our program six years after its implementation. We hypothesized that if the reduction in antibiotic pressure is maintained over time, it could contribute to a decrease in the incidence of nosocomial infections caused by CR-Kp, as well as in the associated mortality rate, variables that were not evaluated in our previous work. 

## 2. Results

Since the start of the ASP, a total number of 4077 face-to-face educational interviews were conducted between the prescribers and the specialists from the antimicrobial stewardship team (see “Intervention” in the Section 4).

### 2.1. Antimicrobial Consumption

**Figure 1:**
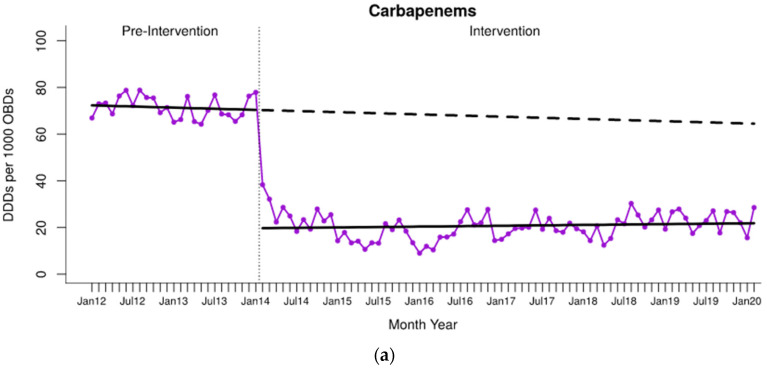

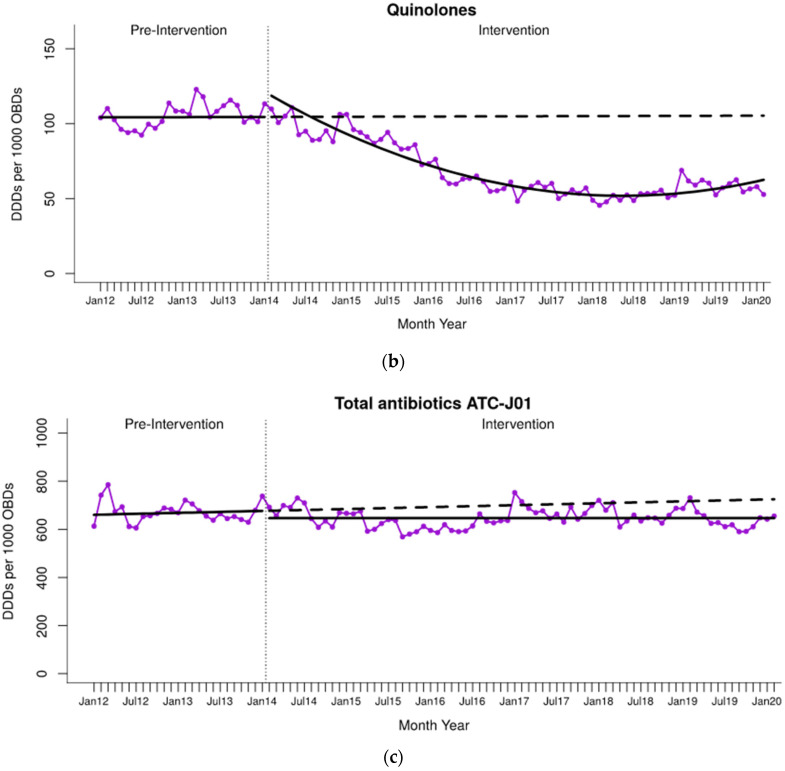
Interrupted time-series analysis of the trends in (**a**) carbapenems, (**b**) quinolones, and (**c**) ATC group J01 (antibacterials for systemic use) consumption observed before and after the implementation of the antimicrobial stewardship program. Solid purple line: antibiotic consumption time series. Solid black lines: observed trend during the pre-intervention and intervention periods. Dashed black line: counterfactual (expected) trend after the intervention according to the pre-intervention values. DDDs, defined daily doses; OBDs, occupied bed days.

The descriptive analysis of monthly antimicrobial consumption is shown in Table S1. Reduced carbapenem consumption was maintained over the long term, with a difference of −66.19% (95% CI −87.03 to −45.34) between the expected pre-intervention trend consumption value and that obtained six years after starting the program (Table 1, Figure 1a). Furthermore, quinolones showed a significant change in trend during the intervention, with a relative decrease in consumption at six years of −40.68% (CI 95% −61.93 to −19.43); meanwhile, the use of the ATC-J01 group remained stable throughout the study period (Table 1, Figure 1b,c).

Regarding the use of β-lactams/β-lactamase inhibitors, although it showed a significant increase after the start of the intervention of 21.25 DDD/1000 stays, this increase was not significantly maintained six years after the start of the intervention. No differences between periods were observed in the consumption of third- and fourth-generation cephalosporins and aminoglycosides at the end of the study (Table 1, Figure S1).

### 2.2. Clinical Outcomes

**Figure 2:**
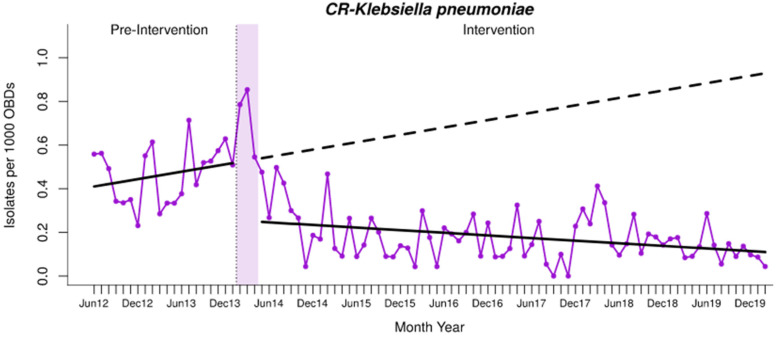
Interrupted time-series analysis of changes in trends in the incidence density of CR-Kp infections observed before and after the intervention. Solid purple line: incidence density of CR-Kp time series. Solid black lines: observed trend during the pre-intervention and intervention periods. Dashed black line: counterfactual (expected) trend after the intervention according to the pre-intervention values. OBDs, occupied bed days.

The descriptive analysis of incidence density (ID) and crude death rates of CR-Kp is shown in Table S2. The ID of CR-Kp decreased significantly and it was maintained over the long term (Table 2, Figure 2), with a relative reduction of −88.14% (95% CI; −100.4 to −75.85) six years after starting the program, compared with the expected values in the absence of intervention, accounting for an absolute reduction of −0.819 cases per 1000 OBDs (−1.634 to −0.003). The proportion of CR-Kp per year is shown in Table 3. 

The crude death rate of hospital-acquired CR-Kp at 14 and 28 days decreased significantly after the intervention. This change did not lead to a significant reduction six years after starting the program (Table 2, Figure S2).

### 2.3. Potential Changes in Healthcare during the Study Period

Indicator trends related to infection control remained stable. Thus, the proportion of hand hygiene compliance did not present any change in trend during the study period (Figure S3) and correct contact isolation was observed in all of the indicated cases. Regarding hospital complexity indicators, the number of major surgical procedures showed a stable trend while the number of ICU OBDs and transplantations increased progressively throughout the study period (Figure S4).

## 3. Discussion

Our results show the long-term positive impact of a mixed ASP to optimize carbapenem use and reduce the incidence of CR-Kp infections in an endemic hospital, confirming the hypothesis that the reduction in antibiotic pressure can contribute to a sustained reduction in the incidence of nosocomial infections caused by these multidrug-resistant microorganisms over time. In addition, the crude death rate related to CR-Kp was significantly decreased after the intervention, remaining steady at the end of the study period.

We previously reported a significant reduction in carbapenem consumption two years after the implementation of PROVAUR [21]. Six years after starting the program, its consumption has been reduced by more than 60% with respect to the value expected in the absence of the intervention, standing below the average level of consumption of all regional hospitals of equal complexity in the same period (21.77 vs. 31.75 DDD/1000 OBDs) [22]. This point seems particularly relevant because the effect of most interventions on ASPs tends to vanish over time, and very few studies report results beyond 2 years [12,16]. On the other hand, many authors have reported successful interventions focused on specific classes of antibiotics, which have shown to be rapid and effective in reducing their consumption and, over time, the specific resistance mechanisms associated with these antimicrobials. However, this approach may lead to a compensatory consumption of alternative antimicrobials (the “squeezing balloon” phenomenon), which may also have even worse effects on bacterial resistance [18,23]. In this study, although our program included a restrictive measure, it did not have a negative impact on overall antimicrobial use, which remained unchanged throughout the study period. In this regard, it should be noted that, in addition to the core activity specifically targeting carbapenem use, our program also implemented other general measures during the intervention period to optimize antimicrobial use. Our results suggest that the sustainability of all these measures allowed us to maintain a lasting benefit on antimicrobial pressure throughout the intervention period.

Regarding the ecological impact of ASPs, recent systematic reviews and meta-analyses have found no strong evidence of the efficacy of these programs in reducing antimicrobial resistance, especially when focusing on specific resistant phenotypes such as CR-Kp [11,12,24]. This is due in part to the complex relationship between antibiotic exposure and the development of resistance. There are mathematical models designed to quantify the relationship between antibiotic consumption and antimicrobial resistance by calculating the threshold of antibiotic use to avoid antibiotic resistance, which could predict the necessary delay for this effect to take place. The THRESHOLD study group has proposed a model that considers interactions between individual- and group-level variables and controls for concurrent factors [25]. Unfortunately, data on carbapenem resistance in Enterobacterales have not yet been included. 

A further impediment in the search for evidence on the efficacy of ASPs in reducing CR-Kp incidence is the limited number of studies evaluating this relationship, many of which are of poor design quality. In a review by Carrara et al. [11] aimed at assessing the impact of ASPs on KPC-Kp rates, after evaluating over 200 eligible papers, only seven studies reported microbiological data. These studies varied in their robustness, from very robust measures (incidence of infections) to less reliable metrics, with no distinction between clinical and screening isolates and no mention of patient days or number of admissions in the denominators. Furthermore, the available studies show great heterogeneity in relation to the study setting, methodology used, and results obtained, which are sometimes contradictory [12,13,14,16]. In this context, although various strategies to optimize carbapenem use have achieved reductions in their consumption, only some studies have found a secondary decrease in CR-Kp, and the evidence for this reduction in the long term is even scarcer. A 4-year multicenter study conducted after the implementation of an educational ASP showed a significant reduction in the rate of carbapenemase-producing Enterobacterales, including KPC-Kp [26]. However, it was limited by the absence of baseline data before the intervention, so any pre-existing time trend could not be excluded. In addition, infections were not distinguished from colonizations in the analysis. In our study, our time-series analysis showed that the ID of CR-Kp infections suffered a significant reduction after the start of the intervention, showing an 88 % lower incidence than expected six years after its implementation. Furthermore, the reduction in the ID of CR-Kp infections was not accompanied by a decrease in the ID of carbapenem-susceptible *K. pneumoniae* infections (Table S4). These data support the idea that the reduction in antibiotic pressure favored by our program may have contributed greatly to this reduction in CR-Kp infections and no other factors should have affected both resistant and susceptible microorganisms equally (e.g., improvements in general preventive or sanitary measures). However, it must be considered that the prevalence of antimicrobial resistance is a result of many other factors, such as infection control practices, exposure to other antimicrobials, and patient comorbidities. For this reason, we analyzed infection control measures and complexity indicators of hospital activity as potential confounders, which remained unchanged except for the number of transplants and number of ICU stays, which increased throughout the study period. We also evaluated the consumption of other antibiotics whose use has been shown to influence KPC-Kp increases [27,28,29]. While the use of cephalosporins and aminoglycosides remained unchanged, quinolone consumption was significantly reduced throughout the post-intervention period. This decrease is likely related to the inclusion of levofloxacin in PROVAUR as part of the package of measures aimed at optimizing antimicrobial use. Considering that the use of quinolones is a potential risk factor for the development of CR-Kp, it is likely that their lower consumption has favored the reduction in these resistant microorganisms. Furthermore, the arrival of ceftazidime–avibactam at our center during the post-intervention period may have contributed to the reduction in the CR-Kp reservoir, although its impact could not be quantified statistically due to the low incidence of use and its unavailability during the entire study period. 

Regarding the clinical impact of ASPs, most studies have only demonstrated the absence of deleterious effects on mortality rates. Other programs have shown significant reductions but are based on exclusively educational measures and were performed in settings without the endemicity of CR-Enterobacterales; so, the impact in highly endemic areas is uncertain [18,30]. In our study, the crude death rate from hospital-acquired CR-Kp decreased significantly after starting the intervention, probably favored by the lower incidence of these infections associated with the reduced use of carbapenems, remaining steady at the end of the study period. The appearance of new effective treatments may also have contributed to this reduction in the mortality rate, as is the case of the arrival of ceftazidime–avibactam in our center during the post-intervention period, which was used in severe cases for the treatment of these infections. The death rate after the diagnosis of global *K. pneumoniae* infections (carbapenem-susceptible and carbapenem-resistant) also decreased after initiation of the intervention (Table S3, Figure S5), probably favored by the reduction in mortality rate observed in CR-Kp infections. 

This study has some limitations. First, we analyzed aggregated data, which precludes the extrapolation of the results to the patient level. Secondly, this is a single-center study, which implies the need to confirm the reproducibility of our findings in other health centers. Thirdly, this is a retrospective and quasi-experimental study. Most of these studies use an uncontrolled before–after design, which is highly susceptible to bias and random error. In our case, we used the interrupted time-series (ITS) methodology, considered the most robust design for assessing the impact of health interventions after clinical trials [31,32]. As a strength of our work, the possible influence of confounding factors throughout the study period was analyzed in detail, taking into account the indicators of hospital complexity, infection control measures, and the use of other antimicrobials that influence the incidence of CR-Kp. Other strengths are the statistical methodology employed and the inclusion of clinical and microbiological outcomes, considering the current need for a greater number of studies that include these outcomes with the aim of generating sufficient useful scientific evidence that can at last solidly demonstrate the clinical and ecological impact of ASPs.

## 4. Materials and Methods

### 4.1. Study Design

A quasi-experimental before-and-after study was performed using ITS based on an ecological time-trend analysis, following the ORION statement. The study period covered 98 months: two years before (1 January 2012 to 31 January 2014) and six years after (1 February 2014 to 29 February 2020) the start of the ASP.

### 4.2. Setting

The study was performed at the Reina Sofia University Hospital (Cordoba, Spain), which is a 1000-bed tertiary-care hospital with 32 intensive care unit (ICU) beds and active transplantation programs. No data from pediatric patients were included. Since June 2012, a high rate of KPC-Kp was identified after an infected patient was transferred from an Italian hospital. The organisms corresponded to ST512 clone and produced KPC-3, in addition to SHV-11 and TEM-1 [20]. The involved strain showed resistance to all tested β-lactams, amikacin, tobramycin, cotrimoxazole, and fluoroquinolones and presented variable susceptibility to tigecycline, colistin, fosfomycin, and gentamicin. The organism was susceptible to ceftazidime–avibactam, although a few isolates resistant to this combination have already been identified [33]. No other hospital outbreaks occurred during the study period. 

### 4.3. Intervention

The methods used have been described elsewhere [21]. In brief, PROVAUR was headed by a multidisciplinary team of 15 Infectious Diseases experts from different clinical units, as well as a hospital pharmacist who identified daily carbapenem prescriptions through the pharmacy’s computerized system, providing the antimicrobial stewardship team with a list of patients. PROVAUR’s main activity was both restrictive (prescription form) and educational through a face-to-face interview between the patient’s attending physician and the specialist from the antimicrobial stewardship team, who jointly reviewed the case and discussed the appropriateness of the prescription and therapeutic alternatives. In addition to the core activity targeting carbapenems, this program also implemented additional measures to optimize antimicrobial use: (1) the implementation of antibiotic consumption indicators in each department; (2) updating local guidelines for the management of empirical treatment, surgical prophylaxis, urinary tract infection, respiratory tract infection, and intra-abdominal infection; (3) monthly training sessions with the PROVAUR team for the healthcare staff to review the most prevalent infectious syndromes in the hospital setting and the main aspects of antibiotic use; (4) the implementation of an intervention program for the duration of antimicrobial treatment (PROVATEM) in all hospitalization units; and (5) the inclusion of levofloxacin in the PROVAUR program. Furthermore, since April 2017, the microbiology service intensified and protocolized the methodology for the detection of carbapenem resistance and characterization of carbapenemases through a comprehensive microbiological diagnostic program. This ASP received institutional support, and its objectives were included in the annual agreement between each clinical unit with the hospital manager and the Andalusian Health Service. No other interventions were performed in the center during the study period.

### 4.4. Study Measures

Antimicrobial use was evaluated through measures of the antibiotic consumption of the Anatomical Therapeutical Chemical (ATC) group J01, carbapenems (meropenem/imipenem), β-lactams/β-lactamase inhibitors, third- and fourth-generation cephalosporins, quinolones, and aminoglycosides. These were recorded as defined daily doses (DDDs) per 1000 occupied bed days (OBDs) and calculated according to the WHO’s ATC Classification System and the 2019 World Health Organization DDD values. The effect of the intervention on the number of nosocomial infections caused by CR-Kp was presented as incidence density (ID) per 1000 OBDs. Every strain was classified as CR or non-CR according to the criteria described below. The impact on mortality rates was assessed as the all-cause crude death rate [34,35] (deaths per 1000 OBDs per month) on days +14 and +28 after the diagnosis of CR-Kp infection. Other variables potentially related to the incidence density of CR-Kp were and monitored. Data on the average proportion of correct hand hygiene compliance in each unit were collected and hand washing was evaluated according to WHO recommendations [36]. The percentage of correct contact isolation in patients colonized/infected with extended-spectrum β-lactamase (ESBL) and CR-GNB was collected following the Centers for Disease Control and Prevention criteria [37]. Indicators related to the complexity of the hospital’s activity were recorded as the number of ICU OBDs, the number of major surgical procedures, and the number of transplants (solid-organ and hematopoietic stem-cell). All the variables were recorded monthly.

### 4.5. Microbiological Studies

*K. pneumoniae* was considered hospital-acquired when it was isolated from a sample obtained ≥48 h after a patient’s admission or in those cases when, even occurring in the first 48 h, the patient was hospitalized or had had contact with the healthcare system during the previous 4 weeks. The recurrent isolation of the same microorganism was considered to represent a unique episode unless the culture was obtained 4 weeks after the last positive sample. Identification and susceptibility testing were initially performed by commercial microdilution panels (MicroScan WalkAway system, Beckman Coulter, Brea, CA, USA). The susceptibility study to new antimicrobials was later completed with Sensitive EURGNCOL panels (ThermoFisher Scientific, Waltham, MA, USA). The identification was confirmed by Maldi-Tof (Bruker Daltonics GmbH, Bremen Germany). Clinical categories were defined according to the corresponding EUCAST breakpoints [38]. ESBL production was confirmed by disk diffusion using disks (Bio-Rad Laboratories, Marnes-la-Coquette, France) of cefotaxime, ceftazidime, and cefepime with and without clavulanic acid in all cases. Carbapenemase production was confirmed using different methods: modified carbapenem inactivation methods (mCIM), rapid colorimetric b-CARBA tests (Bio-Rad, Hercules, CA, USA), commercial PCR assays (Xpert, Carba-R, Cepheid Inc., Sunnyvale, CA, USA, and immunochromatography assays (KPC K-SeT, Coris BioConcept, Gembloux, Belgium and NG-Test CARBA 5, NG-Biotech, Guipry-Messac, France).

### 4.6. Data Analysis

To evaluate the ASP’s effects, we conducted an ITS analysis. We performed a longitudinal segmented regression with a generalized least squares approach to assess changes in level and/or trend after the ASP, adjusting for seasonality and detecting possible outliers. Autocorrelation was considered using autoregressive moving average (ARMA) models. To select the most parsimonious models, we applied the Akaike Information Criterion. The normality of residuals was verified, and the autocorrelation patterns were validated through likelihood ratio tests to determine the final model for each variable. To evaluate the long-term effect attributable to the ASP, we calculated absolute and relative differences between the values of the expected pre-intervention trend and the modeled trend at the end of the study. To take into account the delay between the effect of antimicrobial use and resistance, we considered a 3-month phase-in period [39] in the ITS analysis of the incidence density of *K. pneumoniae*. Statistical analysis was performed using R software, version 3.6.1. A Joinpoint regression analysis [40] (Joinpoint Regression program, National Cancer Institute, US government, version 4.7.0) was performed to detect significant trend changes associated with the intervention on indicators of hospital complexity. Confidence intervals (CI95%)or p-values were included to indicate statistical significance. Differences were considered statistically significant at *p* < 0.05 (2-tailed tests).

### 4.7. Ethics Approval

The study was conducted in accordance with the Declaration of Helsinki, and the protocol was approved by the Ethics Committee of the University Hospital Reina Sofia (approval number 5339).

## 5. Conclusions

In conclusion, the results of this study suggest that the implementation of an educational and restrictive ASP contributed to decreasing the high incidence of infections and mortality rates of CR-Kp, achieving a sustained ecological and clinical effect. Further quality evidence needs to be generated through appropriately designed studies and outcome measures to further promote ASPs and clarify which interventions are most successful for controlling the spread of CR-Kp.

## Figures and Tables

**Table 1:** Interrupted time-series analysis of changes in trends in antimicrobial consumption.

Outcomes	Regression Intercept	Pre-Intervention Trend	Change in Level ^a^	Change in Trend ^b^	Absolute Effect ^c^	Relative Effect (%) ^c^
**Total antibiotics** **(ATC-J01)**	659.3	0.667 (−2.642, 3.976)	−30.009 (−78.72, 18.70)	−0.663 (−4.175, 2.849)	−78.39 (−362.8, 206)	−10.82 (−45.92, 24.29)
**Carbapenems**	72.39	−0.081 (−0.510, 0.349)	−50.691 (−56.95,−44.43)	0.109 (−0.349, 0.569)	−42.68 (−79.66, −5.69)	−66.19 (−87.03, −45.34)
**Third- and fourth-generation cephalosporins**	80.15	0.416 (−0.442, 1.274)	0.347 (−10.94, 11.63)	−0.319 (−1.302, 0.664)	−22.92 (−98.41, 52.56)	−18.95 (−70.16, 32.24)
**β-lactams and β-lactamase inhibitors**	185.5	−0.342 (−1.635, 0.952)	21.25 (1.86, 40.64)	0.746 (−0.634, 2.127)	75.72 (−35.8, 187.3)	49.8 (−59.28, 158.8)
**Quinolones**	104.1	0.01 (−0.419, 0.442)	16.74 (10.01, 23.47)	−2.65 (−3.022, −2.288)	−42.86 (−79.92, −5.79)	−40.68 (−61.93, −19.43)
**Aminoglycosides**	21.35	−0.167 (−0.487, 0.153)	1.121 (−3.827, 6.069)	0.155 (−0.179, 0.489)	12.421 (−15.05, 39.89)	248.2 (−1645, 2142)

Data are presented as monthly defined daily doses (DDDs) per 1000 occupied bed days (OBDs) with a 95% confidence interval unless otherwise specified. ^a^ Increase or decrease in the first month after the start of the intervention period with respect to the expected value. ^b^ Change in slope for the intervention period. ^c^ Absolute or percentage difference between the expected value according to the pre-intervention trend in antibiotic prescription and the trend six years after the start of the intervention.

**Table 2:** Interrupted time-series analysis of changes in trends in incidence and mortality rate of CR-Kp.

Outcomes	Regression Intercept	Pre-Intervention Trend	Change in Level ^a^	Change in Trend ^b^	Absolute Effect ^c^	Relative Effect (%) ^c^
**Incidence density**	0.404	0.006 (−0.004, 0.015)	−0.284 (−0.434, −0.135)	−0.008 (−0.017, −0.002)	−0.819 (−1.634, −0.003)	−88.14 (−100.4, −75.85)
**14-day crude death rate**	0.097	0.001 (−0.003, 0.005)	−0.066 (−0.129, −0.002)	−0.001 (−0.006, 0.003)	−0.164 (−0.515, 0.186)	−85.28 (−115.1, −55.39)
**28-day crude death rate**	0.110	0.002 (−0.003, 0.007)	−0.084 (−0.160, −0.007)	−0.003 (−0.008, 0.002)	−0.302 (−0.722, 0.119)	−88.85 (−105.3, −72.36)

Data are presented as monthly incidence density and all-cause crude death rate per 1000 occupied bed days with a 95% confidence interval unless otherwise specified. ^a^ Decrease in the first month after the start of the antimicrobial stewardship program (ASP) period with respect to the expected value. ^b^ Change in slope for the ASP period. ^c^ Percentage difference between the expected value according to the pre-intervention trend and the trend six years after the start of the ASP.

**Table 3:** Proportion of carbapenem-resistant *Klebsiella pneumoniae* (CR-Kp) infections per year.

Year	Number of Kp Infections	Number of CR-Kp infections	Resistance Proportion (%)
2012 *	140	64	45.7%
**2013**	188	121	64.4%
**2014**	205	118	57.6%
**2015**	138	47	34.1%
**2016**	266	45	16.92%
**2017**	260	32	12.30%
**2018**	259	54	20.85%
**2019**	284	35	12.32%
2020 **	36	3	8.3%

* Data for 2012 correspond only to the months included in the study period: June to December. ** Data for 2020 correspond only to the months included in the study period: January and February.

## Data Availability

The data presented in this study are available upon request from the corresponding author.

## Supplementary Materials

The following supporting information can be downloaded at https://www.mdpi.com/article/10.3390/antibiotics13090792/s1: Figure S1: Interrupted time-series analysis of changes in consumption of other antibiotics; Figure S2: Interrupted time-series analysis of changes in trends in the crude death rates of CR-*K. pneumoniae*; Figure S3: Joinpoint regression analysis of hand hygiene compliance; Figure S4: Joinpoint regression analysis of complexity indicators associated with healthcare during the study period; Figure S5: Interrupted time-series analysis of changes in trends in the incidence and mortality rates of Global-*K. pneumoniae*; Table S1: Descriptive analysis of antimicrobial consumption; Table S2: Descriptive analysis of incidence and mortality rate of carbapenem resistant-*K. pneumoniae*; Table S3: Interrupted time-series analysis of changes in trends in the incidence and mortality rates of Global-*K. pneumoniae*; Table S4: Interrupted time-series analysis of changes in trends in the incidence and mortality rates of carbapenem-susceptible *K. pneumoniae.*
