# Supplementary material for: Long-Term Clinical and Ecological Impact of an Antimicrobial Stewardship Program on the Incidence of Carbapenem-Resistant Klebsiella pneumoniae Infections in a High-Endemic Hospital
Source: Antibiotics (Basel). 2024 Aug 23;13(9):792. doi: 10.3390/antibiotics13090792 (PMC11429328; doi:10.3390/antibiotics13090792)
Supplement: Supplementary file 1 [file antibiotics-13-00792-s001.zip › antibiotics-3146456-supplementary.pdf]

Supplementary material

Supplementary Figure S1. **Interrupted time-series analysis of changes in consumption of other antibiotics**

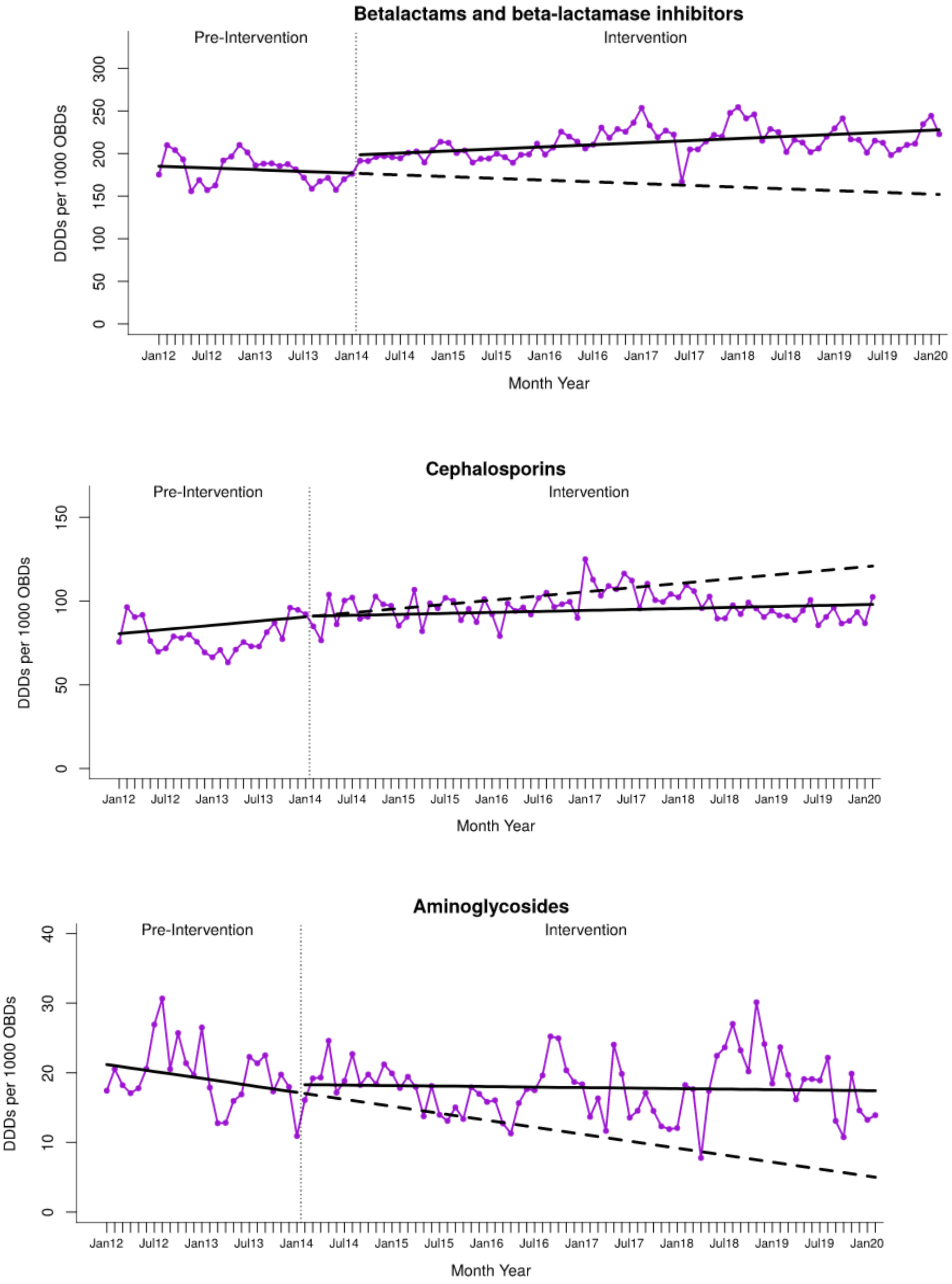

Date are presented as monthly defined daily doses (DDD) per 1000 occupied bed days (OBDs).
Solid purple line: antibiotic consumption time series. **Solid black lines**: pre-intervention and intervention trends.
Dashed black line: counterfactual (expected) trend.

Supplementary Figure S2*. Interrupted time-series analysis of changes in trends of crude death rate of carbapenem resistant-*K. pneumoniae

a) **14-day crude death rate**

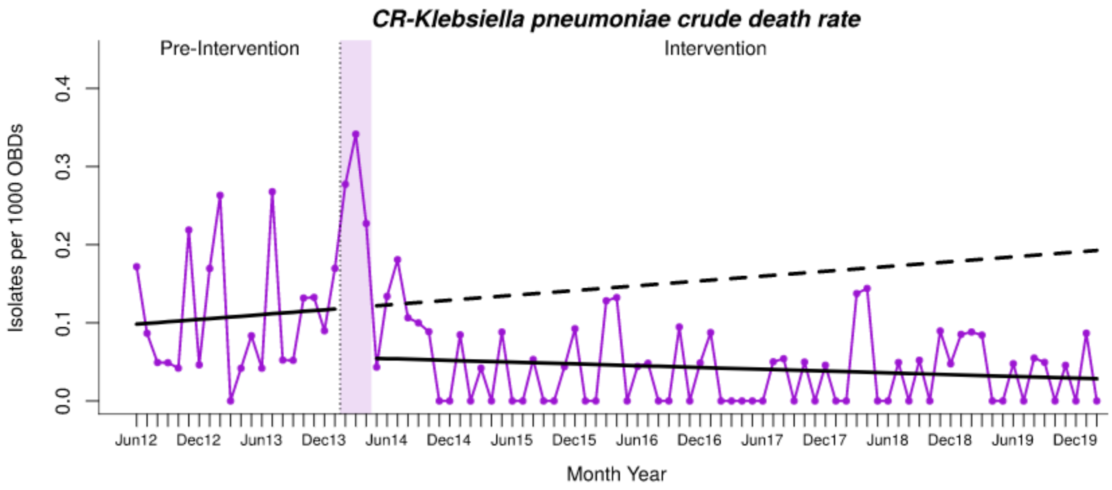

b) **28-day crude death rate**

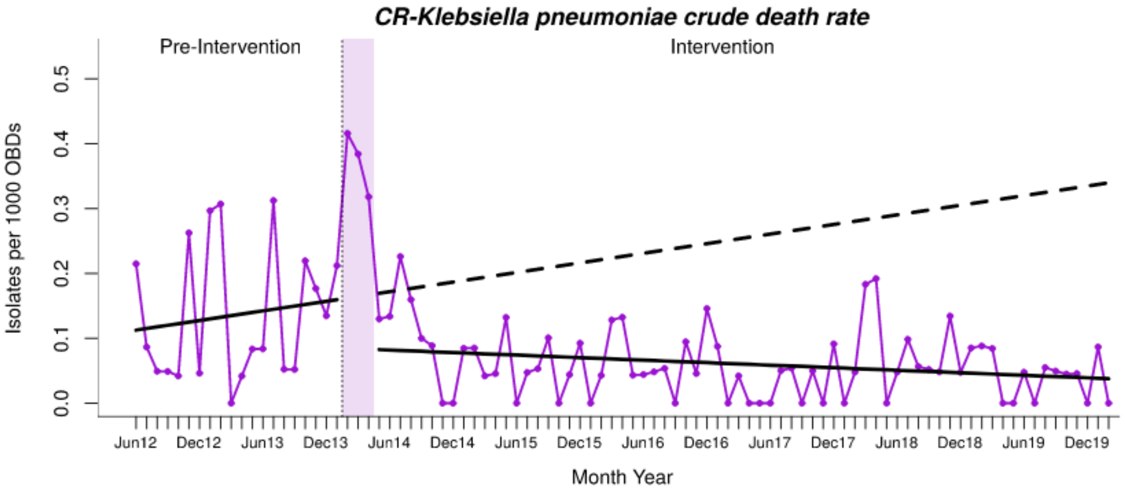

Date are presented as monthly isolates per 1000 occupied bed days (OBDs).
Solid purple line: CR-*Klebsiella pneumoniae* crude death rate. **Solid black lines**: pre-intervention and intervention trends. **Dashed black line**: counterfactual.

Supplementary Figure S3. **Joinpoint regression analysis of hand hygiene compliance**

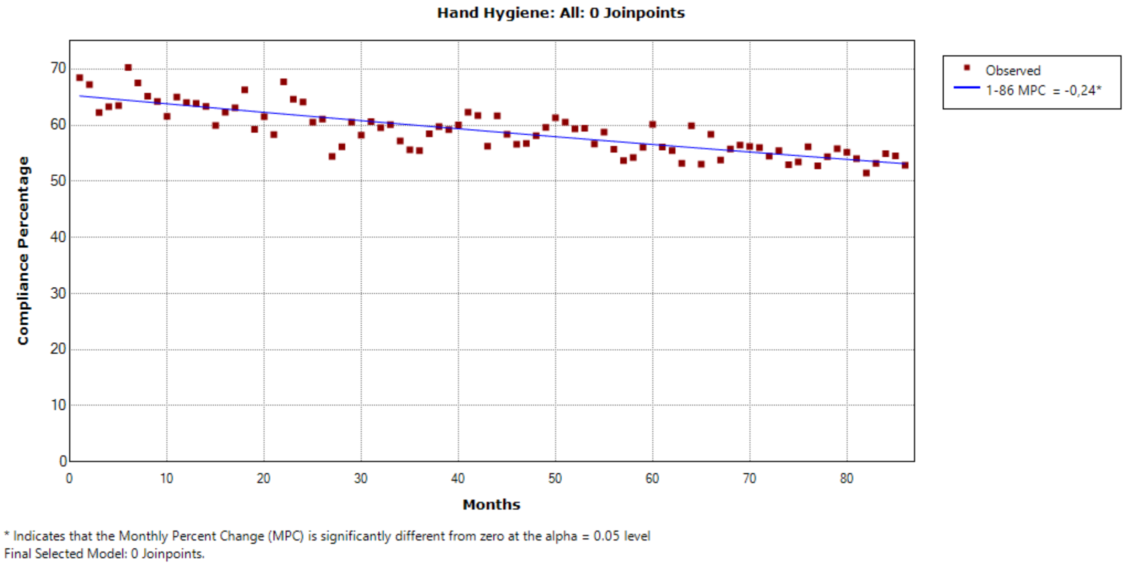

Supplementary Figure S4. **Joinpoint regression analysis of complexity indicators associated with healthcare during the study period**

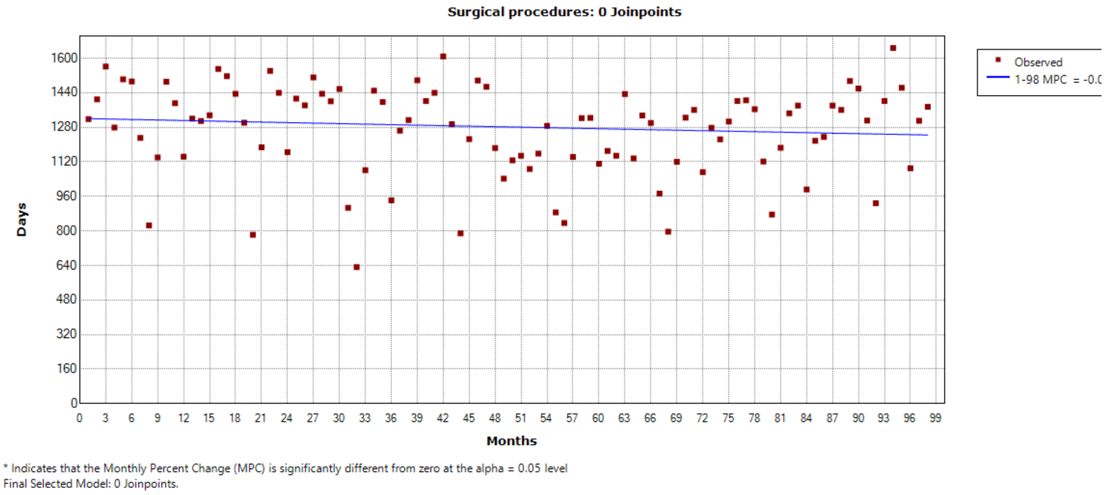

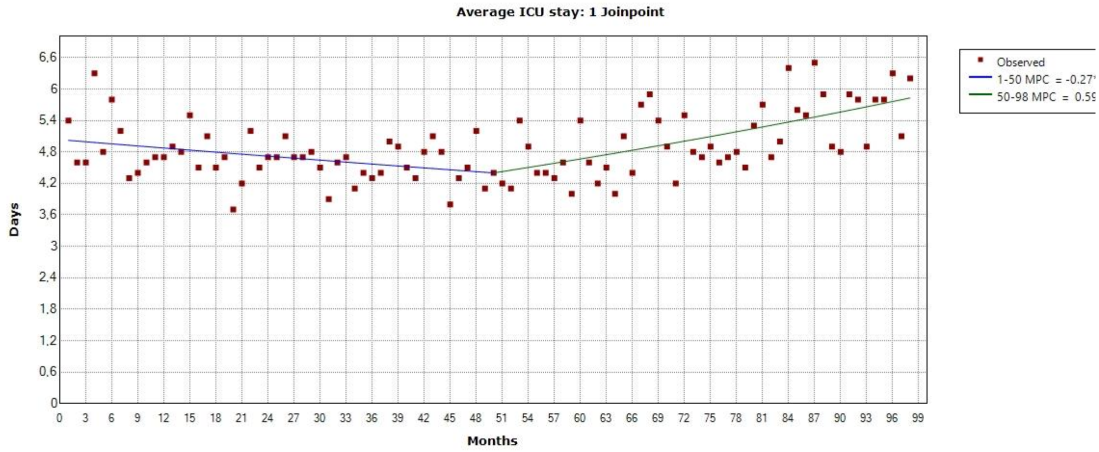

* Indicates that the Monthly Percent Change (MPC) is significantly different from zero at the alpha = 0.05 level
Final Selected Model: 1 Joinpoint.

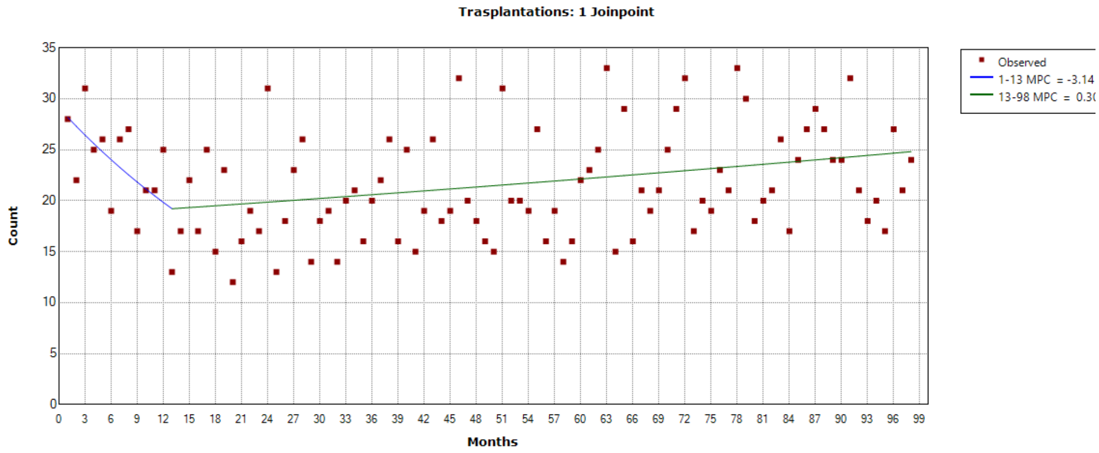

* Indicates that the Monthly Percent Change (MPC) is significantly different from zero at the alpha = 0.05 level
Final Selected Model: 1 Joinpoint.

Supplementary Figure S5. Interrupted time-series analysis of changes in trends of incidence and mortality rate of Global-K. pneumoniae

a) Incidence density

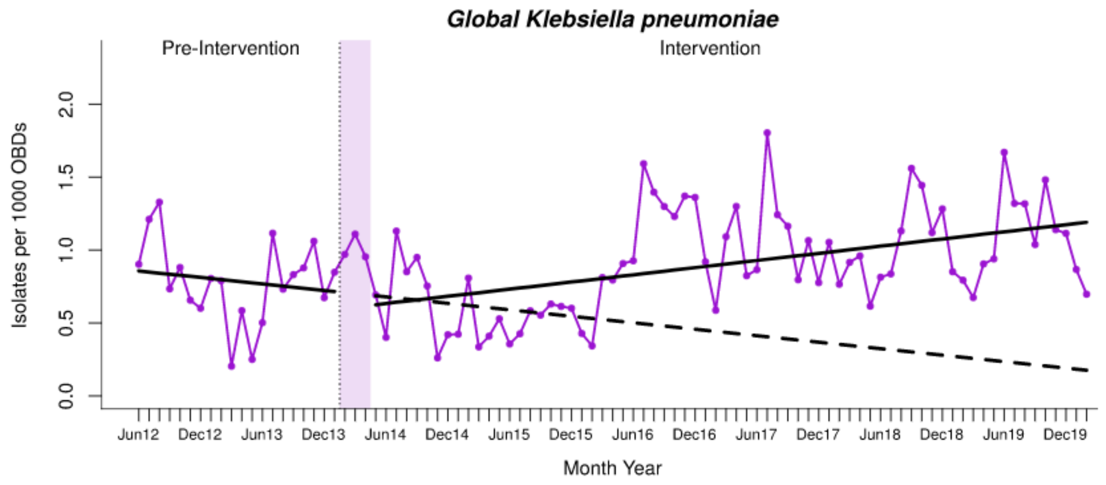

b) 14-day crude death rate

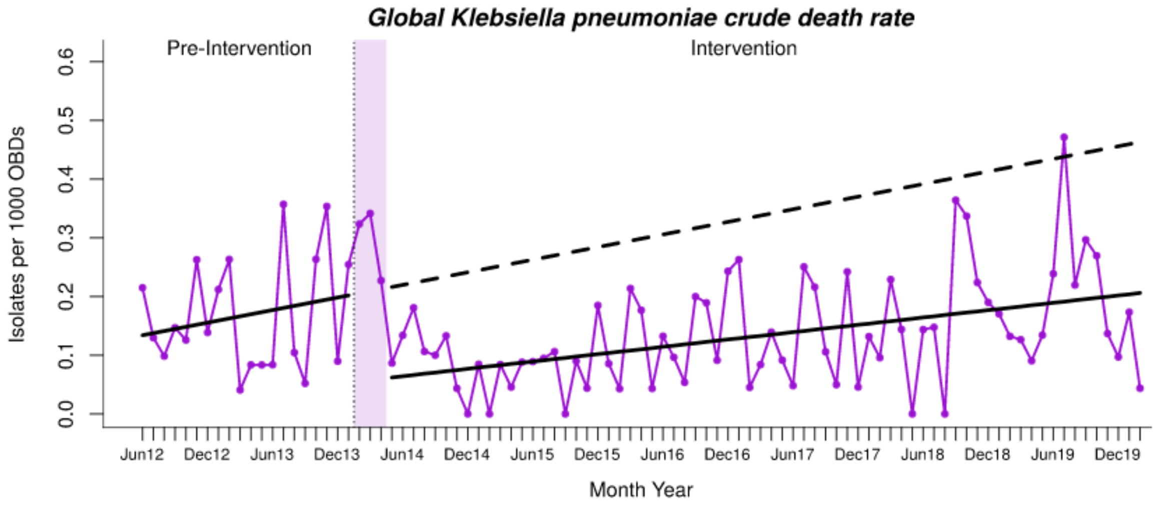

c) 28-day crude death rate

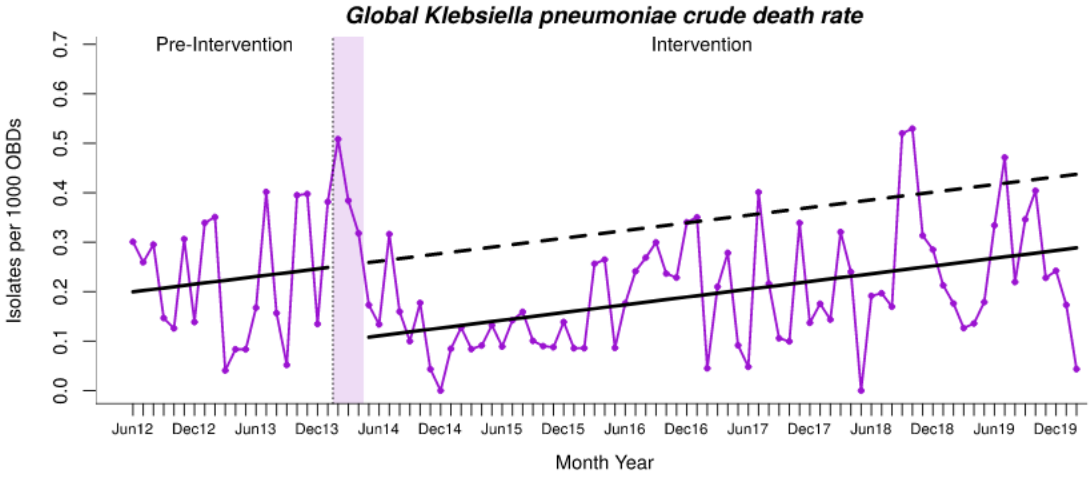

Date are presented as monthly isolates per 1000 occupied bed days (OBDs).
Solid purple line: global *Klebsiella pneumoniae* crude death rate. **Solid black lines**: pre-intervention and intervention trends. **Dashed black line**: counterfactual.

Supplementary Table S1. Descriptive analysis of antimicrobial consumption

Year	2012	2013	2014	2015	2016	2017	2018	2019	2020*
**Total antibiotics (ATC-J01)**	669.5 (643.0-689.5)	659.6 (643.4-677.5)	679.9 (641.5-701.3)	618.1 (592.1-645.9)	616.6 (595.2-633.5)	672.6 (658.6-693.8)	652.8 (634.1-681.1)	625.9 (610.9-660.0)	648.2 (645.0-651.3)
**Carbapenems**	73.1 (70.8-75.8)	68.3 (65.4-71.7)	25.2 (22.7-29.5)	14.2 (13.4-18.6)	16.5 (13.8-22.1)	19.5 (18.5-20.5)	21.1 (17.4-23.8)	23.5 (20.4-26.7)	22.1 (18.8-25.3)
**Third- and fourth-generation cephalosporins**	77.0 (74.7-82.6)	74.3 (70.9-82.8)	94.7 (88.5-100.8)	95.5 (88.3-100.4)	96.3 (92.1-98.8)	108.1 (102.5-112.4)	96.6 (91.8-102.4)	91.2 (88.6-94.2)	94.6 (90.7-98.5)
**β-lactams and β-lactamase inhibitors**	192.5 (167.3-202.1)	176.6 (169.2-186.5)	195.9 (191.6-201.3)	198.8 (194.1-201.4)	219.2 (209.7-226.5)	220.9 (211.9-228.6)	218.0 (211.2-232.0)	213.9 (208.8-219.9)	233.6 (228.2-239.0)
**Quinolones**	100.6 (96.0-105.1)	108.3 (104.4-113.1)	98.0 (91.8-107.1)	88.4 (85.3-94.2)	62.2 (58.9-64.2)	56.5 (53.4-58.7)	51.4 (48.8-53.3)	59.5 (56.0-61.9)	55.3 (54.0-56.6)
**Aminoglycosides**	20.5 (18.1-22.5)	17.9 (16.7-21.6)	19.0 (18.0-20.1)	17.4 (13.9-18.0)	17.6 (15.8-19.8)	14.5 (13.3-17.4)	21.3 (17.6-23.8)	19.0 (15.8-19.7)	13.6 (13.4-13.7)

Data are presented as median (interquartile range) of monthly-defined daily doses (DDDs) per 1000 occupied bed days (OBDs).

* Data for 2020 correspond only to the months included in the study period: January and February.

Supplementary Table S2. Descriptive analysis of incidence and mortality rate of carbapenem resistant-K. pneumoniae

Year	2012*	2013	2014	2015	2016	2017	2018	2019	2020**
**Incidence density**	0.35 (0.34-0.53)	0.52 (0.37-0.58)	0.45 (0.27-0.52)	0.14 (0.09-0.22)	0.18 (0.12-0.23)	0.10 (0.08-0.17)	0.19 (0.14-0.29)	0.14 (0.09-0.15)	0.07 (0.05-0.08)
**14-day crude death rate**	0.05 (0.05-0.13)	0.09 (0.05-0.14)	0.12 (0.08-0.19)	0.02 (0.00-0.06)	0.02 (0.00-0.06)	0.00 (0.00-0.05)	0.02 (0.00-0.06)	0.05 (0.00-0.06)	0.04 (0.02-0.06)
**28-day crude death rate**	0.05 (0.05-0.15)	0.11 (0.05-0.24)	0.15 (0.10-0.25)	0.05 (0.04-0.09)	0.05 (0.04-0.10)	0.02 (0.00-0.05)	0.05 (0.05-0.11)	0.05 (0.00-0.06)	0.04 (0.02-0.06)

Data are presented as median (interquartile range) of monthly incidence density and monthly all-cause crude death rate per 1000 occupied bed days.

*Data for 2012 correspond only to the months included in the study period: June to December.

** Data for 2020 correspond only to the months included in the study period: January and February.

Supplementary Table S3. Interrupted time-series analysis of changes in trends of incidence and mortality rate of Global-K. pneumoniae

Outcomes	Regression intercept	Pre-intervention Trend	Change in Level[a]	Change in Trend[b]	Absolute Effect[c]	Relative effect (%)[c]
**Incidence density**	*0.864*	-0.007 (-0.041, 0.026)	-0.076 (-0.601, 0.448)	0.015 (-0.019, 0.049)	1.014 (-1.799, 3.828)	576.66 (-10205.6, 11359)
**14-day crude death rate**	0.130	0.003 (-0.001, 0.008)	-0.152 (-0.215, -0.090)	-0.001 (-0.006, 0.003)	-0.257 (-0.644, 0.129)	-55.51 (-92.59, -18.43)
**28-day crude death rate**	0.197	0.002 (-0.004, 0.009)	-0.151 (-0.243, -0.059)	0.000 (-0.007, 0.007)	-0.149 (-0.720, 0.422)	-34.018 (-120.15, 52.11)

Data are presented as monthly incidence density and all-cause crude death rate per 1000 occupied bed days with a 95% confidence interval, unless otherwise specified. **a** Increase or decrease in the first month after the start of the antimicrobial stewardship program (ASP) period with respect to the expected value. **b** Change in slope for the ASP period. **c** Percentage difference between the expected value according to the pre-intervention trend and the trend six years after the start of the ASP.

Supplementary Table S4. Interrupted time-series analysis of changes in trends of incidence and mortality rate of carbapenem susceptible-K. pneumoniae

Outcomes	Regression intercept	Pre-intervention Trend	Change in Level[a]	Change in Trend[b]	Absolute Effect[c]	Relative effect (%)[c]
**Incidence density**	*0.503*	-0.021 (-0.053, 0.012)	0.291 (-0.026, 0.609)	0.029 (-0.005, 0.064)	2.437 (-0.260, 5.135)	-172.30 (-305.29, -39.30)
**14-day crude death rate**	0.045	0.001 (-0.003, 0.005)	-0.053 (-0.109, 0.004)	0.001 (-0.003, 0.005)	0.027 (-0.318, 0.373)	19.11 (-266.1, 304.3)
**28-day crude death rate**	0.089	-0.0002 (-0.007, 0.007)	-0.052 (-0.160, 0.057)	0.003 (-0.004, 0.010)	0.168 (-0.439, 0.775)	245.1 (-2805.9, 3296.3)

Data are presented as monthly incidence density and all-cause crude death rate per 1000 occupied bed days with a 95% confidence interval, unless otherwise specified. **a** Increase or decrease in the first month after the start of the antimicrobial stewardship program (ASP) period with respect to the expected value. **b** Change in slope for the ASP period. **c** Percentage difference between the expected value according to the pre-intervention trend and the trend six years after the start of the ASP.
